# Diagnosis in Scrapie: Conventional Methods and New Biomarkers

**DOI:** 10.3390/pathogens12121399

**Published:** 2023-11-28

**Authors:** Diego Sola, Marina Betancor, Paula A. Marco Lorente, Sonia Pérez Lázaro, Tomás Barrio, Eloisa Sevilla, Belén Marín, Bernardino Moreno, Marta Monzón, Cristina Acín, Rosa Bolea, Juan J. Badiola, Alicia Otero

**Affiliations:** 1 Centro de Encefalopatías y Enfermedades Transmisibles Emergentes, Facultad de Veterinaria, IA2, Universidad de Zaragoza, 50013 Zaragoza, Spain; 683728@unizar.es (D.S.);; 2 Unité Mixte de Recherche de l’Institut National de Recherche pour l’Agriculture, l’Alimentation et l’Environnement1225 Interactions Hôtes-Agents Pathogènes, École Nationale Vétérinaire de Toulouse, 31076 Toulouse, France

**Keywords:** scrapie, prion diseases, PrPSc, prion biomarkers

## Abstract

Scrapie, a naturally occurring prion disease affecting goats and sheep, comprises classical and atypical forms, with classical scrapie being the archetype of transmissible spongiform encephalopathies. This review explores the challenges of scrapie diagnosis and the utility of various biomarkers and their potential implications for human prion diseases. Understanding these biomarkers in the context of scrapie may enable earlier prion disease diagnosis in humans, which is crucial for effective intervention. Research on scrapie biomarkers bridges the gap between veterinary and human medicine, offering hope for the early detection and improved management of prion diseases.

## 1. Prion Diseases

Prion diseases are a group of transmissible neurodegenerative diseases with a long incubation period and an invariably fatal course. These diseases affect various species, including humans. They are caused by the post-translational conversion of the cellular prion protein (PrP^C^), a glycoprotein physiologically present in mammals and encoded by the *PRNP* gene, into its anomalous isoform called PrP^Sc^. Following this conformational change, PrP^Sc^ acquires partial resistance to protease digestion, insolubility in non-ionic detergents, and a high resistance to both physical and chemical sterilization processes, as well as a tendency to form aggregates [1,2]. In animals, examples of these processes are scrapie in sheep and goats, bovine spongiform encephalopathy (BSE), and chronic wasting disease (CWD) in cervids, among others. In the human species, we find Creutzfeldt–Jakob disease (CJD), with its sporadic (sCJD), iatrogenic (iCJD), and familial (fCJD) forms; a variant of CJD (vCJD); fatal familial insomnia (FFI); Gerstmann–Sträussler–Scheinker syndrome (GSS); variable protease-sensitive prionopathy (VPSPr); and kuru [3].

These diseases are also known as transmissible spongiform encephalopathies (TSEs), since the main neuropathological feature they present is vacuolization, or spongiform degeneration, of the central nervous system (CNS), which can be observed with the appearance of vacuoles both intraneuronal (vacuolization) and in the neuropil (spongiosis). In addition, other neuropathological findings are gliosis, neuronal loss, and PrP^Sc^ deposition in the CNS [4,5]. These lesions result in clinical manifestations of neurodegenerative signs in the host including behavioral alterations and motor ataxia. The incubation period is long, and clinical signs may take months to years to appear, although once they appear, the course is rapid and invariably leads to death of the individual [2,6].

Depending on their origin, prion diseases can be classified as acquired, genetic, or sporadic. Acquired diseases are those that appear as a result of infection by the causative agent, the prion protein. Genetic or familial forms are those produced by the existence of mutations in the *PRNP* gene, which produce abnormal forms of the prion protein that have a greater predisposition to undergo pathological conformational changes. Finally, sporadic or spontaneous TSEs are those that have no known apparent cause. Most animal TSEs are infectious in nature, i.e., acquired. In humans, however, sporadic TSEs are the most frequent, accounting for about 85% of diagnosed cases [7].

Prion diseases share similarities with other neurodegenerative processes such as Alzheimer’s disease (AD), Parkinson’s disease (PD), Amyotrophic Lateral Sclerosis (ALS), or Huntington’s disease (HD), which are also caused by the accumulation of protein aggregates in the CNS and are, therefore, included under the concept of proteinopathies [8]. The prion protein and those causing the aforementioned diseases share characteristics regarding their propagation, but despite the fact that some of these pathological proteins are infective under experimental conditions, PrP^Sc^ is able to not only replicate its conformation but also to be transmitted between individuals under natural conditions [9].

## 2. Scrapie

Scrapie is a naturally occurring prion disease affecting goats and sheep [2,10,11]. Two types of scrapie are currently known: classical scrapie and atypical scrapie. Classical scrapie is the oldest known TSE, having been first described in the United Kingdom in 1732. Since then, it has spread throughout most of the world and is endemic in many countries. It is also considered the archetype of TSEs, serving as a model for understanding other TSEs. Actually, the letters “Sc” in the term PrP^Sc^ (widely used to refer to the prions causing TSEs) come from the word scrapie (PrP^Sc^ = scrapie prion protein) [11,12]. On the other hand, atypical scrapie was first discovered in Norway in 1998, although retrospective studies show its existence at least since 1972 [13]. Furthermore, it is also present worldwide, even in countries considered free of classical scrapie, such as Australia and New Zealand [11,14].

Classical and atypical scrapie differ in their pathogenesis and transmission. Classical scrapie affects goats and sheep from 2 to 5 years of age, and its infectious nature is well known; it is a highly heterogeneous disease as it can be caused by several different prion strains [15]. On the other hand, atypical scrapie is caused by a single strain (Nor98), which usually affects goats and sheep older than 5 years; atypical scrapie cases seem to be sporadic in nature, although several studies have demonstrated the infectious character of this strain [15,16,17].

Regarding interindividual transmission, classical scrapie can be naturally acquired by several routes. The main one is the horizontal route, either by direct contact between animals or indirectly through contamination of the environment with prions released through the excretions, secretions, semen, placental debris, or carcasses of infected animals [18,19,20,21]. Placentas are considered to be the main source of contamination, as they contain very high infective titers [22].

Other possibilities include the vertical or maternal route, although uncertainties exist regarding the timing and route of transmission, as it can occur during gestation (intrauterine transfer) or, most frequently, during delivery (via contamination from the placenta) or in the postnatal period (by the ingestion of infected colostrum or milk as well as via contact with a contaminated environment) [20,23,24]. Cases of iatrogenic transmission through the use of injections, blood transfusions, or other biological products from scrapie-infected animal tissues have also been described [18].

Under natural conditions, prions enter the organism mainly by the oral route, as a result of ingestion of contaminated material from affected individuals and later uptake at the level of the intestine, although uptake at the level of the palatine tonsil has also been described [25,26]. Classical scrapie can also be naturally transmitted through lesions in the skin and mucous membranes [27,28]. Other routes have also been proven successful in experimental conditions, such as intracerebral, intraperitoneal, intravascular, intraocular, intranasal, and conjunctival [18,20,29].

Ingested prions pass through the digestive tract and cross the intestinal epithelium thanks to the action of digestive enzymes (which fragment PrP^Sc^, favoring its transport in association with other proteins), dendritic cells (as they capture antigens from the intestinal lumen), and M cells, a type of specialized epithelial cell located in the gut-associated lymphoid tissue (GALT) of ileal Peyer’s patches [30,31,32]. M cells play a key role in the uptake and transport of antigens from the intestinal lumen to the subepithelial lymphoid tissue, where they present the antigens to macrophages and follicular dendritic cells of the lymphoreticular system (LRS) [31,33,34].

Once in the LRS, the immune response and the replication and accumulation of PrP^Sc^ within macrophages and follicular dendritic cells continues for some time. The extent of lymphoid replication varies depending on the prion strain involved and the host genotype, with most classical scrapie agents showing a strong lymphotropism, which allows for an early diagnosis of the disease through prion detection in biopsies of palatine tonsils [35], third eyelids [36], or rectal mucosa [37]. In contrast, lymphotropism remains very limited in animals with genotypes resistant to classical scrapie [30,38].

Neuroinvasion usually begins when prions in the enteric LRS are transferred to the enteric nervous system, from which they spread to the CNS via peripheral nerves or by the hematogenous route [31]. Once in the CNS, PrP^Sc^ disseminates throughout the brain and spinal cord, as well as to other peripheral organs or tissues. PrP^Sc^ accumulation triggers a neurotoxic response characterized by neuroinflammation, synaptic alterations, neuronal death, and neuropil vacuolization or spongiform neuronal degeneration which, after an incubation period of several years, manifest as a set of neurological symptoms that eventually lead to the animal’s death 1 to 6 months after the onset [39].

In comparison with classical scrapie, the natural transmission and pathogenesis of atypical scrapie is poorly documented. PrP^Sc^ accumulation and spread are restricted to the CNS, thus supporting the sporadic nature of the pathology [11]. However, the presence of infectivity has been proven in the lymphoid tissues, nerves, and muscles of individuals with natural or experimental atypical scrapie, even though PrP^Sc^ is not detected [40]. This, added to studies demonstrating the possibility of the experimental oral transmission of the pathology, has led to a rethinking of the etiology of the disease [40].

## 3. Diagnosis of Scrapie

### 3.1. Scrapie as an Animal Health Problem: Diagnosis in the Context of Epidemiological Surveillance Programs

Scrapie is listed in the Terrestrial Code of the WOAH (formerly OIE) as an infectious disease affecting sheep and goats whose control and eradication must be pursued. It has been a compulsorily notifiable disease in all European Union (EU) member states since 1993. Following the “mad cow disease” crisis that shook Europe in the 1990s, TSE-control measures were reinforced, and scrapie, together with bovine spongiform encephalopathy, was subjected to a strict monitoring plan aimed at preventing potentially dangerous prions from entering the human food chain.

The current EU monitoring system includes passive surveillance, i.e., testing of clinically suspicious animals, and active surveillance, i.e., testing of a number of apparently healthy animals slaughtered for human consumption and of animals found dead.

Passive surveillance remains one of the pillars of the scrapie monitoring system in the EU [41]. It relies on the identification, usually by the farmer or by field veterinarians, of clinically affected animals. This clinical diagnosis requires a certain expertise and, within the legal framework of scrapie monitoring, needs confirmation by laboratory techniques. Clinical signs of classical scrapie are usually observed in animals aged 2 to 5 years, and the onset is usually insidious, starting with changes in mental status, activity, and behavior that eventually give way to more obvious neurological signs such as pruritus, muscle tremors, a loss of body condition or weight, ataxia, and gait incoordination, which will eventually lead to weakness, lateral decubitus, and, finally, complete prostration. On the other hand, the signs of atypical scrapie usually appear in animals older than 5 years, the main one being ataxia in the absence of pruritus. In addition, other signs such as circling, changes in behavior and posture, tremors, visual impairment, and a loss of body condition may occur [42,43].

As already stated, clinical diagnosis is not useful in preclinical stages, since the onset of signs usually occurs in advanced stages. Therefore, both passive and active surveillance programs eventually rely on the use of laboratory techniques that allow for the confirmation of clinically diagnosed animals or the detection of infected individuals among the sampled, apparently healthy animals, respectively [44]. For the diagnosis to be definitive, it must be made post-mortem from CNS samples, as false negatives can occur with lymphoid tissues. At present, there is still no fully effective method for antemortem diagnosis.

For a long time, the laboratory diagnosis of scrapie was mainly based on histopathological analysis and the observation of CNS samples for lesions associated with prions, such as vacuolization, gliosis, and neuronal degeneration and loss, or amyloidosis [12,45,46,47,48]. The lesions observed in classical and atypical scrapie are similar, although they differ in the distribution pattern, with classical scrapie showing a bilateral and symmetrical distribution in the spinal cord, brainstem, and hypothalamus, whereas in atypical scrapie, vacuolization occurs more frequently in the cerebellar and cerebral cortexes and in the basal ganglia, being absent in the brainstem [12,46].

However, the histopathological analysis usually requires the support of other laboratory tests, since the absence of observable lesions in the CNS does not always imply the absence of diseases, and, oppositely, finding these alterations does not always imply an infection with prions (i.e., histopathological lesions are not pathognomonic of prion disease). Currently, most diagnostic techniques are based on the immunological detection of PrP^Sc^ accumulation in tissue samples, mainly the CNS, as accumulation of PrP^Sc^ is specific to TSEs and occurs before the appearance of lesions. This technique is even useful for diagnosis from autolytic and frozen tissues or when lesions are minimal or absent [49].

Among these techniques, the gold standard methods Western blot and immunohistochemistry (IHC) are particularly noteworthy. Both are based on subjecting tissue samples to proteolytic digestion with proteinase K (PK) for a subsequent detection of the PK-resistant fragment (PrP^res^) of PrP^Sc^ using specific antibodies [49]. In addition to providing a definitive diagnosis, both techniques have some additional perks.

On the one hand, Western blot allows for the characterizing of prion strains and for discerning between classical and atypical scrapie according to the electrophoretic pattern, which varies among strains in the relative proportion of glycoforms and in the sites of proteolytic cleavage. Specifically, in classical scrapie, three bands corresponding to unglycosylated, monoglycosylated, and diglycosylated proteins are observed [16,49,50,51], whereas in atypical scrapie, five bands can be visualized [16].

On the other hand, IHC is also useful to differentiate classical and atypical scrapie since it allows for the in situ detection of PrP^Sc^ deposits, whose cellular localization, tissue distribution, and morphological characteristics vary with the prion strain and the host genotype [49,52]. Specifically, in classical scrapie, the immunostaining observed is usually bilateral, mainly localized in medulla oblongata, and shows various morphological patterns of deposition, both intracellular (intraneuronal and intraglial) and extracellular (stellate or glial, perivascular, subpial, subependymal, linear, fine punctate, coarse punctate, coalescent, perineuronal, and plaque-like) [48,52]. By contrast, PrP^Sc^ deposition in the brainstem of atypical scrapie cases is rare, appearing in cerebellar and cerebral cortices. In addition, a morphological pattern of intraneuronal deposition is not detected, but others, such as fine and coarse stippling, are detected [16].

It is worth noting that IHC also allows for the detection of PrP^Sc^ in lymphoid tissues associated with the third eyelid, palatine tonsils, or rectal mucosa [53], thus providing a reliable tool for antemortem diagnosis and even the identification of individuals in preclinical stages of the disease by an analysis of biopsies of easily accessible tissues [35,36,37]. However, the sensitivity of this method is variable, as it depends on the extent of prion distribution in the lymphoid tissues, which varies with the genotype and prion strain, and this is very limited in sheep with disease-resistant genotypes [54,55,56] and in atypical scrapie [11].

Other tests based on PrP^Sc^ immunodetection (usually in brain samples) are the so-called “rapid tests” such as ELISA (enzyme-linked immunosorbent assay), which are also of great importance for screening, i.e., an analysis of large numbers of animals in a considerably short time due to their speed and simplicity, which makes them key tools in scrapie surveillance and eradication programs. In addition, these techniques have proven a certain potential for antemortem diagnosis, although this is not definitive and still requires diagnostic confirmation (by Western blot or IHC) in the event of a positive or inconclusive result [56].

### 3.2. Scrapie as a Model for Human Prion Diseases: The Search for Diagnostic Biomarkers

Despite the efforts that the EU has put into controlling scrapie in member states (which have adopted the highest-level legal framework in the world for the protection of consumers against TSEs), the disease remains an enzootic pathology in most parts of the European territory. The disease frequently passes unnoticed when not actively looked for; in addition, its relatively low economic impact and its non-zoonotic nature have held back resolute attempts at scrapie eradication. On the other hand, the small ruminant livestock sector mobilizes much less money than other livestock sectors such as cattle or pork; given their lower economic value, the investment that the farmers and the society are willing to make in individualized diagnoses is lower, and, therefore, the highly sensitive, state-of-the-art diagnostic techniques are not applicable in this context.

Biomarkers are measurable indicators that can provide information about the presence, progression, or severity of a disease. In prion diseases, biomarkers can be various molecules or changes in the brain or elsewhere that reflect the pathological processes associated with the accumulation of abnormal prion proteins. Since the beginning of the BSE food crisis, considerable efforts have been made to develop sensitive methods to detect the causative agent of TSEs and to search for other markers of the disease, in order to control the spread of these diseases in both humans and animals.

In sheep, it is possible to diagnose scrapie in preclinical stages by the immunohistochemical detection of PrP^Sc^ in lymphoid tissues obtained through biopsies [35,36,57,58,59]. The possibility of identifying scrapie-infected animals before the onset of overt clinical signs makes scrapie a convenient model of preclinical diseases in humans. Preclinical sheep can be detected on the basis of the presence of PrP^Sc^ deposits in gut-associated lymphoid tissues (GALTs) obtained by rectal biopsies. Tissues and biological fluids obtained from these preclinical individuals can thus be analyzed to confirm the efficacy of commonly used biomarkers for the diagnosis of prion disease in preclinical stages. They can even serve in the search for novel biomarkers, for instance using discovery proteomics, that would later be confirmed in studies with humans.

To date, in humans, prion diseases can only be identified and diagnosed in symptomatic patients [60]. At the moment these diseases are identified, the neuronal damage is already massive, and, therefore, possible treatments would have a limited benefit. In this context, scrapie, in which preclinical animals are easy to detect using a rectal biopsy [37], might represent a good model for evaluating early-stage biomarkers for prion diseases.

In human prion diseases, the biopsy of brain tissue allows for in vivo diagnosis by observing the characteristic neuropathological changes of the disease (spongiosis, neuronal loss, accumulation of PrP^Sc^, and gliosis) [61] and by studying the molecular profile of the responsible prion by Western blot [62]. However, the increasing sensitivity and accuracy of less invasive diagnostic techniques, such as the cerebrospinal fluid (CSF) biomarker analysis, have rendered brain biopsy obsolete. It is currently only employed in cases where the differential diagnosis includes other treatable nervous disorders that cannot be distinguished from a prion disease by other means [63]. Therefore, nowadays, the clinical diagnosis of human prion diseases relies on the analysis of a series of biomarkers in CSFs. At the same time, these analyses allow for the exclusion of other nervous disorders for which treatment exists, unlike prion diseases. Although the cerebrospinal fluid is used, there are other even less invasive samples such as blood or nasal swabs which are gaining importance in recent years [64,65].

## 4. Biomarkers in Prion Diseases

TSE biomarkers can be classified into three groups. On the one hand, we find the prion protein and its derivatives, which are directly involved in pathogenesis. On the other hand, there are “surrogate” markers, which are proteins released into the ventricular system, blood, or other biological fluids due to neuronal death and neurodegeneration. Moreover, in recent years, new studies concerning genetic biomarkers, especially microRNAs, have pointed them out as a new possibility to explore regarding the diagnosis of TSEs.

### 4.1. Prion Protein and Its Derivatives

This group includes the analysis of the total PrP in CSFs and the direct detection of the causal agent (the prion). Regarding the measurement of the total PrP in CSFs, several studies have shown a decrease in the total PrP in the CSF of Creutzfeldt–Jakob disease patients [66,67,68]. The reason for the decrease in the total PrP in the CSF of prion cases is unknown, but it is suggested to be a consequence of its accumulation and aggregation in the brain tissue, which makes it less available in the CSF.

On the other hand, the direct detection of the causal agent (the prion) is challenging due to the low levels of PrP^Sc^ in CSFs and since it requires the use of amplification techniques. These techniques include protein misfolding cyclic amplification (PMCA) and real-time quaking-induced conversion (RT-QuIC).

PMCA is based on the nucleation–polymerization replication process of PrP^Sc^. In this technique, a small amount of PrP^Sc^ (the “seed”) is incubated, along with an excess of PrP^C^ from healthy brains (the “substrate”), and is subjected to serial cycles of incubation and sonication [69]. During the incubation phase, the seed, which is an oligomer of PrP^Sc^, recruits excess PrP^C^ molecules present in the substrate, leading to the growth of the amyloid fiber. In the sonication phase, the fiber fragments, giving rise to numerous new seeds that replicate the process cyclically. In this manner, the amount of PrP^Sc^ generated de novo increases exponentially over several successive cycles of incubation and sonication.

The second of the in vitro prion amplification techniques is called “real-time quaking -induced conversion” (RT-QuIC). This technique was developed from the union of two other known methodologies [70]: the QuIC (quaking-induced conversion) method, in which a PrP^Sc^ seed is amplified on a PrP^C^ recombinant protein in a similar way to PMCA but by shaking [71], and the ASA (amyloid seeding assay) method, which detects the fluorescence emitted by thioflavin in the presence of amyloid [72]. Thus, RT-QuIC monitors the progressive amplification of PrP^Sc^ through the fluorescence emitted by thioflavin. The analysis of the total PrP and PrP seeding activity in CSFs has been assessed in classical scrapie, with results supporting the use of these markers for the detection of this disease. In a study carried out in sheep [73], these markers were analyzed in the CSF from clinical and preclinical scrapie-infected sheep compared to non-infected sheep. In the mentioned study, and in agreement with the results found in sCJD cases, they observed decreased PrP levels (total PrP quantification) as well as seeding activities (RT-QuIC) in the CSF of animals infected with classical scrapie. Moreover, these features were not only significant in clinical animals but also in preclinical animals. Thus, they demonstrated that PrP-related CSF tests can be useful even for the identification of preclinical scrapie cases. The results concerning preclinical sheep in this study were in agreement with previous studies that had detected positive PrP seeding activities by RT-QuIC in the CSF of intracerebral scrapie-infected hamsters before the onset of clinical signs [74]. Numerous authors have demonstrated that the RT-QuIC technique, when applied to CSF samples from human patients, was effective for diagnosis, as it possessed a higher specificity than that of the other “surrogate” biomarkers used to date [70,75]. In addition, this technique has been used to detect seeding activities in other samples obtained from patients. Therefore, samples of nasal brushings [64] and skin [76,77] have demonstrated to be as or more efficient than CSF samples for the diagnosis of CJD cases. Other samples, such as blood [65] and tear fluids [78], although showing seeding activities and being less invasive to obtain, contain fewer prions. This technique has also been successful in the diagnosis of CWD using different biological samples such as third eyelids [79], skeletal muscles [80], saliva, urine [81], and feces [82].

### 4.2. Surrogate Biomarkers

Synaptic degeneration is an early pathogenic event in many neurodegenerative diseases and has been especially reported in prion diseases [39,83]. Because of this, one of the most studied strategies in the search for preclinical biomarkers of TSEs is the study of molecules related to synapse degeneration. This group includes the 14-3-3 protein, total tau, and the light chain neurofilament protein (NfL), which are considered markers of neuronal damage and are routinely analyzed [63,84,85,86]. There are other markers of neuronal death that can complement the diagnosis of prion diseases, such as α-synuclein [73,87], S100b protein [88], glial fibrillary acidic protein (GFAP), neuron-specific enolase (NSE) [63], and neurogranin [89], but their diagnostic efficacy is not fully established.

#### 4.2.1. 14-3-3 and Total Tau

14-3-3 and tau are both considered markers of neuronal damage [84] and, therefore, can be detected in the CSF of several neuropathologies. A study assessing the 14-3-3 and total tau in sheep CSFs [73] showed that these proteins can be used as markers for classical scrapie, even in the preclinical stages of the disease. These surrogate markers of neuronal damage were found significantly increased in sheep CSFs at the clinical stages of the disease. Moreover, even though no statistical significance was found, the markers were also increased in the CSF of preclinical sheep compared to controls. These findings support the use of 14-3-3 and total tau as CSF biomarkers in classical scrapie and suggest that the underlying neurodegenerative mechanisms that release these proteins to the CSF also occur in the early stages of neurodegeneration, therefore pointing at them as possible biomarkers for the preclinical stages of prion disease.

A wide variety of studies have shown that CSF detection of the 14-3-3 protein can be used as a differential diagnostic marker for CJD with good sensitivity and specificity [86,90,91]. Regarding total tau, this protein has also been detected in the CSF of CJD patients, even with a better ability to predict disease stages than 14-3-3. Moreover, both proteins have been found to be correlated [92]. Combining both biomarkers allows for differentiating human prion diseases from other neurodegenerative processes, and, therefore, the combination of the surrogate markers 14-3-3 and total tau in CSFs is currently the routine analysis for the diagnosis of human prion disease cases.

#### 4.2.2. Neurogranin (Ng)

Neurogranin (Ng) is a neuronal postsynaptic protein that is specifically localized in the neuronal soma and dendrites [93]. Ng has been studied in clinical and preclinical scrapie-infected animals compared to healthy controls, in order to elucidate its possible use as a preclinical biomarker for prion diseases. A decrease in Ng expression (both at the protein and gene level) in the CNS in clinical animals compared to controls was detected, in agreement with previous results obtained in sCJD cases [94]. Moreover, significant reductions in Ng expression in the CNS of preclinical scrapie-affected sheep as compared with negative controls were also described, and clinical and preclinical animals showed very similar levels of Ng expression. Currently, there are no effective tools to measure Ng in ovine CSFs. The development of these tests would be of great interest.

This protein has also been studied as a marker of neuronal damage in AD and sCJD patients [89], and it has been observed that its levels in the CSF of sCJD patients are increased with respect to healthy controls and even AD cases. While Ng levels in CSFs were higher in clinical sCJD, the Ng expression in the brain tissue was decreased, therefore resulting in a negative correlation between the protein concentrations in brain tissues and CSFs, which would reflect a potential mobilization of this protein from the CNS into the CSF. Furthermore, in sCJD cases, the concentration of Ng in the CSF does not differ between the early and late stages of the disease, supporting the use of Ng as a biomarker to detect synaptic dysfunction in the early stages of prion disease. These findings, together with those described for scrapie, support the measurement of Ng CSF levels to diagnose human and animal prion diseases, also pointing to Ng as a possible preclinical marker.

#### 4.2.3. Neurofilament Light Chain (NfL)

Like synaptic degeneration, a loss of axonal function occurs in the early stages of prion diseases [39]. Neurofilaments (Nfs) are cytoskeleton proteins located mainly in axons, where they act by conferring structural stability [95]. Neurofilament light chain (NfL) is the most widely used biomarker related to neurodegeneration, as when neuronal damage occurs, this protein is released into the interstitial fluid, which communicates with the CSF and blood. NfL has been seen to increase in the blood and CSF in a wide range of neurological disorders, such as AD, ALS, frontotemporal dementia (FTD), or multiple sclerosis (MS) [96]. Concerning scrapie, the previously mentioned study comparing the levels of Ng in clinical and preclinical scrapie-infected sheep compared to negative controls also assessed the levels of NfL in the CNS and CSF of these animals [94]. As for Ng, NfL levels were lower in the CNS of scrapie-infected sheep, both in the clinical and preclinical group. In this occasion, NfL could also be measured in the CSF of the animals, showing a negative correlation with its levels in the CNS. Thus, a significant increase in the levels of NfL was found in the CSF of clinical scrapie-infected sheep compared to negative controls. Although not statistically significant, preclinical infected sheep also showed higher levels of CSF NfL than controls. Moreover, NfL was measured in the serum of a cohort of preclinical sheep and was found to be increased compared to negative controls [97]. Therefore, although further studies may be needed to confirm its usefulness, recently, NfL has stood out as an interesting biomarker to take into account for the early diagnosis of prion diseases.

NfL has also been found to increase in the CSF and blood of clinical cases of sCJD [98,99,100].

#### 4.2.4. YLK-40

Microglial and astrocyte cell activation is a key feature in many neurodegenerative diseases. Prion diseases are disorders that do not present a typical immune or inflammatory response [101], and it has not been elucidated whether the activation of these mechanisms contributes to the neurodegeneration process or appears as a consequence of the pathogenesis of these diseases [102]. However, neuroinflammation phenomena have been widely reported in these disorders [103], and, therefore, the potential of glial activation and neuroinflammation biomarkers in prion diseases has also been assessed. Among the markers of inflammation and astroglial activation studied, YKL-40 glycoprotein is the most promising. Increased levels of YKL-40 mRNA have been observed in murine models of scrapie in the whole brain of MLR-infected animals in the late preclinical and clinical stages of the disease [104]. The YKL-40 levels of CSF have been shown to be increased in sCJD with respect to other neurodegenerative diseases [104]. Recently, this glycoprotein was quantified in plasma from different patients with neurodegenerative diseases, highlighting the higher levels in patients with CJD [105].

#### 4.2.5. Antichymotyipsin (α1-ACT)

α1-antichymotrypsin (ACT), a serine protease inhibitor protein, is closely related to Alzheimer’s pathology [106], as it was found to be elevated in the CSF of patients with Alzheimer’s disease [107], as it has been proven to exist in the amyloid deposits that are the hallmark of this neuropathology. Although this protein has not been studied in natural cases of scrapie, an increased expression of the gene encoding this protein has been detected in the brains of scrapie-infected mice at preclinical stages [108]. Moreover, the levels of the α1-ACT protein have also been reported to be increased in the brain of mice infected with three different scrapie strains, 263K-, 139A-, and ME7, also displaying a time-dependent progression during the infection [109]. This fact is in accordance with the elevation of the α1-ACT protein in AD due to the accumulation of amyloid deposits, although in prion diseases, no direct colocalization between PrP and α1-ACT has been found [109]. Although it has been limitedly reported in prion diseases, it has been described to be dramatically increased in the urine of patients suffering from sCJD disease and also to progressively increase throughout the course of the disease [108].

Finally, an increased excretion of α1-ACT has also been described in different animal TSEs [110]. Due to all this evidence, and under the need for further studies concerning natural scrapie cases, α1-ACT could play an important role as a biomarker for scrapie and other prion diseases, especially when measured in the urine.

### 4.3. Genomic Biomarkers

In recent years, genomic tools, such as the use of microarrays [111] or massive sequencing of the transcriptome [112,113], have been developed. These tools make it possible to compare global gene expression levels between samples from diseased and healthy individuals, or between different stages of the disease, being able to identify the genes involved in prion pathogenesis that may serve as potential biomarkers for the diagnosis of the disease or as targets for use in therapeutic strategies [114]. Genomic expression studies in CNS samples from both individuals affected by natural diseases, including sheep scrapie, and TSE-adapted murine models have so far identified a large number of differentially transcribed genes, which points to widespread alterations in several pathways of neuronal function, such as cell cycle, cell death, and stress response, thus contributing to the understanding of the molecular pathways involved in the pathogenesis of TSEs [115,116,117,118,119,120,121].

MicroRNAs (miRNAs) have become widely recognized lately as potential diagnostic biomarkers of neurodegenerative diseases [122]. These are small non-coding RNA molecules of around 22 nucleotides in length that regulate post-transcriptional gene expression and protein synthesis by binding to messenger RNAs. Their secretion by affected tissues into body fluids, such as the CSF and blood, and their high stability in them due to chaperone-binding and exosome protection allow for a minimally invasive diagnosis [123]. Alterations in miRNAs have been frequently observed in several neurodegenerative diseases, which is to be expected given that more than half of the protein-coding genes are regulated by miRNAs [124]. Additionally, they have been suggested to play a key role in neuronal survival and homeostasis, as well as in neuroinflammation [125,126].

Regarding scrapie, miRNAs in murine models have been analyzed in the last few years, finding interesting miRNA deregulations in the preclinical stage of diseases, before the onset of clinical signs, although a considerable change of their expression during disease progression has been observed [127,128]. MicroRNA changes have also been reported in the CSF and plasma of sheep, the natural model of scrapie [129,130]. Because of all these findings over the last few years in the microRNA investigation in neurodegenerative disorders, these molecules have a great potential to be used as diagnostic biomarkers.

Numerous miRNAs have been found to be dysregulated in the blood and CSF of AD patients, regulating the metabolism of the characteristically aggregated proteins that deposit in the CNS of these patients and the neuroinflammation and neuronal apoptosis, becoming crucial for the onset and progression of AD pathology [131,132]. Specifically, in prion diseases, although their modulation has not been as well investigated, variations in miRNA expressions have been also observed. Common altered miRNAs have been detected among AD and sCJD patients, suggesting potential common pathogenic mechanisms, both in the CNS [73] and in the blood [133]; despite their similarities, some of these miRNAs have been proven useful to discriminate between both diseases [133]. miRNAs have also been investigated in CWD [134].

In conclusion, current diagnostic techniques for prion diseases are of limited effectiveness. The only pathognomonic biomarker of the disease is the presence of PrP^Sc^ in the nervous system, which can only be demonstrated post-mortem. Consequently, there is a need for the identification of new biomarkers, which are measurable in readily available tissues and fluids, such as the blood or CSF, and which allow for the diagnosis of individuals in the preclinical stage of the disease. Biomarker research in scrapie may, therefore, allow for an earlier diagnosis of human prion disease, which is crucial for effective intervention, offering hope for the early detection and better management of prion diseases.

## Data Availability

Data are contained within the article.
